# Supplementary material for: Surface-Available HER2 Levels Alone Are Not Indicative of Cell Response to HER2-Targeted Antibody–Drug Conjugate Therapies
Source: Pharmaceutics. 2024 Jun 2;16(6):752. doi: 10.3390/pharmaceutics16060752 (PMC11206718; doi:10.3390/pharmaceutics16060752)
Supplement: Supplementary file 1 [file pharmaceutics-16-00752-s001.zip › pharmaceutics-3029890-supplementary.pdf]

Supplemental Information

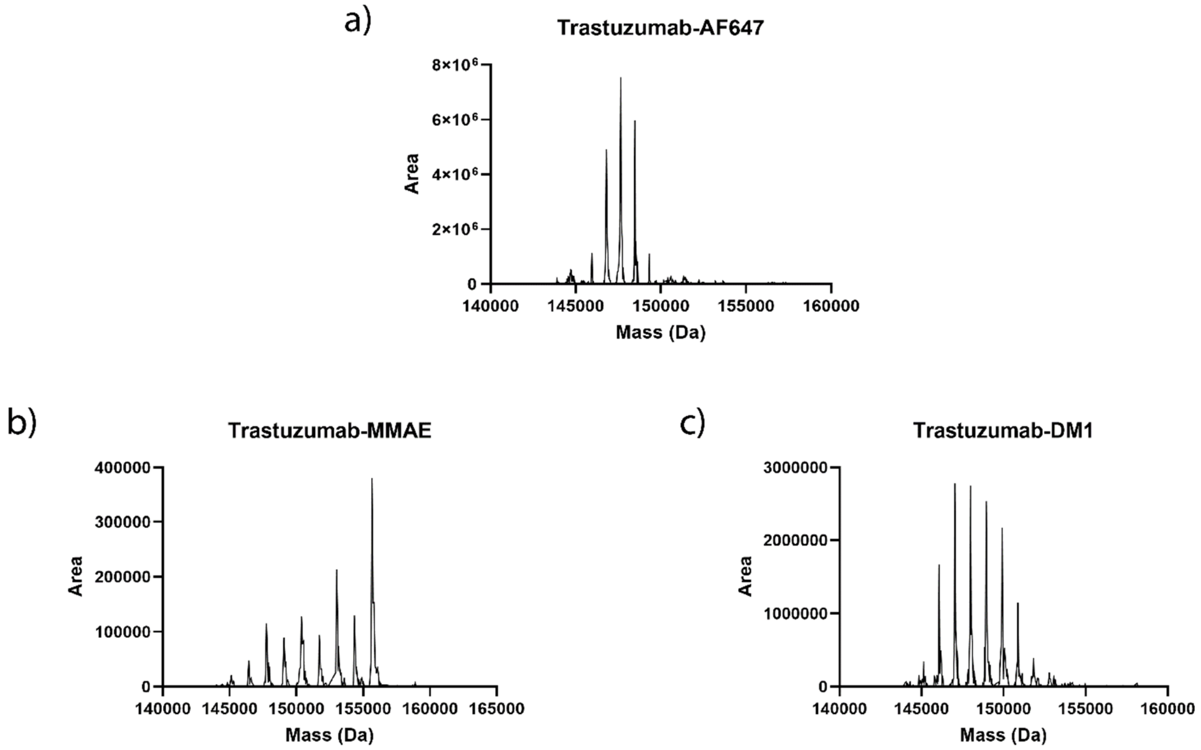

Figure S1. Mass spectrometry spectra of trastuzumab conjugates. Electrospray ionization (ESI) was the method for these spectra; see the methods section for additional mass spectrometry conditions. a) Trastuzumab-AF647 spectrum, fluorophore to antibody ratio (FAR) determined to be 3.2. b) Trastuzumab-MMAE spectrum, drug-to-antibody ratio (DAR) determined to be 5.6. c) Trastuzumab-DM1 spectrum, DAR determined to be 3.4.

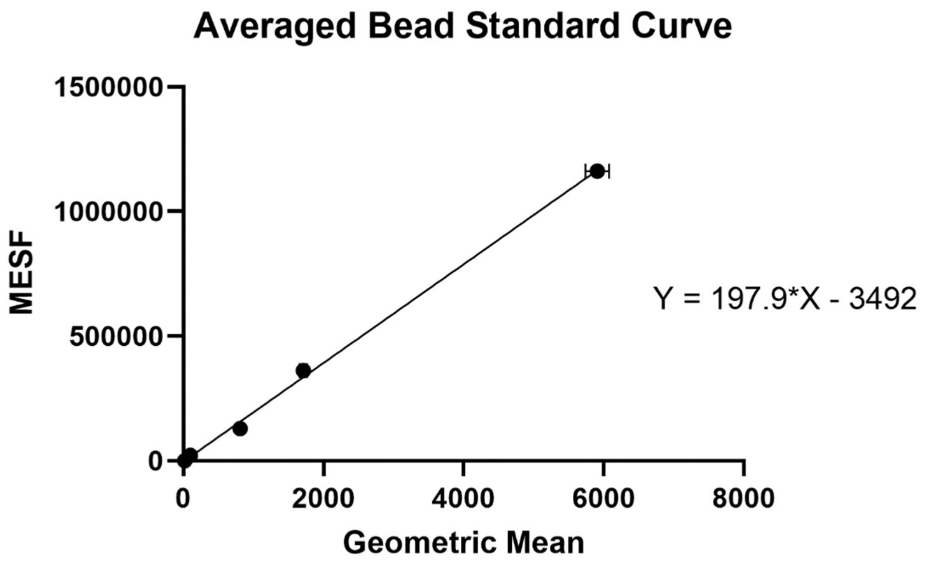

Figure S2. Calibration of molecules of equivalent soluble fluorochrome (MESF), indicating the number of fluorophores, with fluorescence detected from flow cytometry of microspheres (Bang Laboratories). Geometric Mean values shown were averaged from independent experiments, N = 8. Calibration curves used for cellular HER2 quantitation calculations were determined on the day of the experiment. Curve fit was generated in GraphPad Prism 10.2.1.

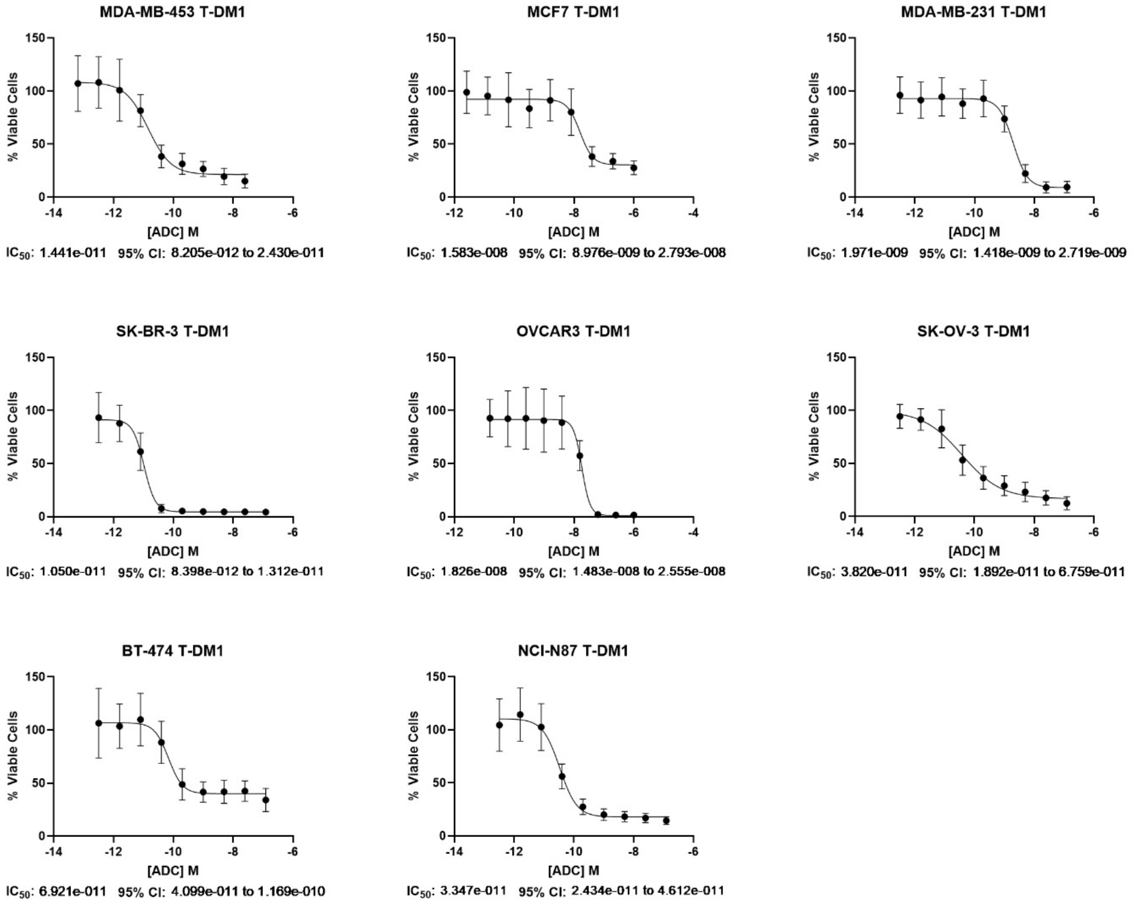

Figure S3a. Viability curves of all cell lines upon treatment with T-DM1. N = 9. IC_{50} values of the cytotoxicity for ADC were determined for normalized data using logistic non-linear regression analysis with GraphPad Prism software version 10.2.1. Curve fits were constrained as needed to a bottom of greater than 0% viability and a top of less than 100% viability.

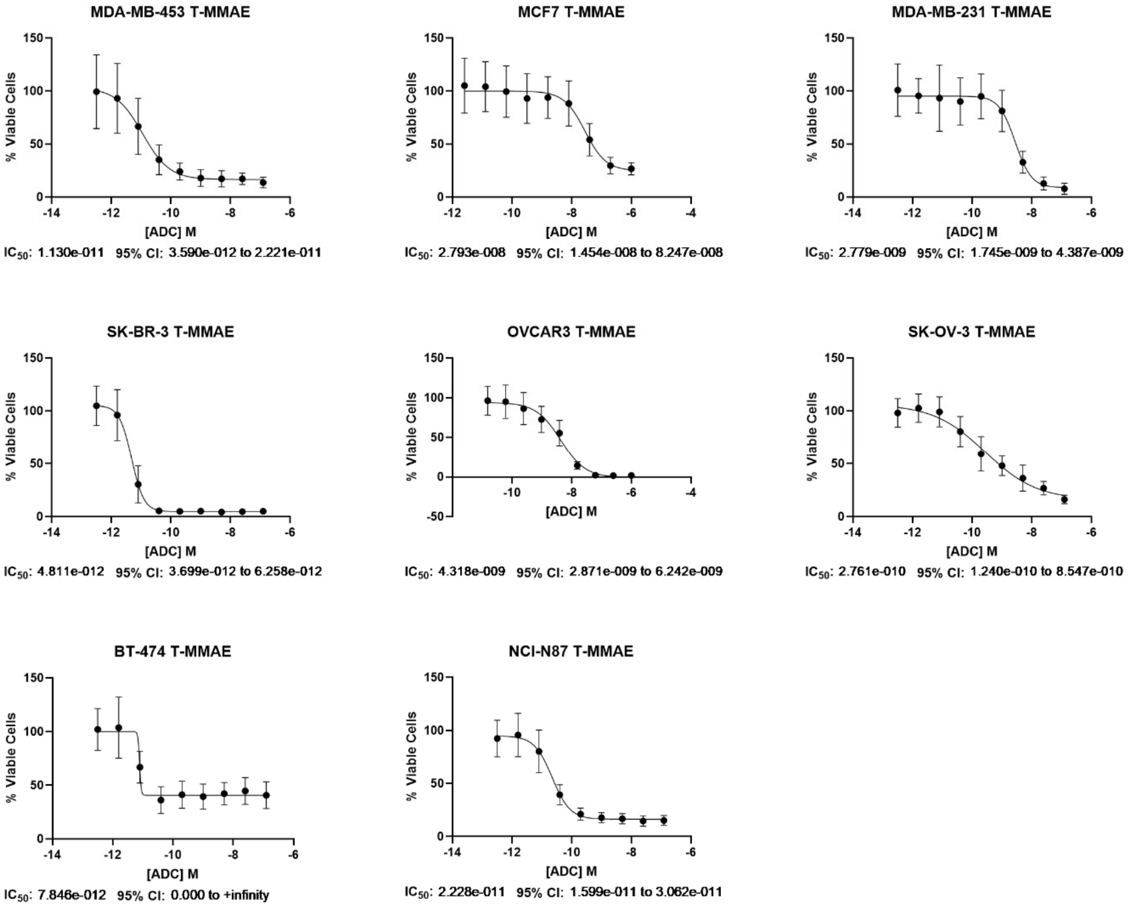

Figure S3b. Viability curves of all cell lines upon treatment with T-MMAE. N = 9. IC_{50} values of the cytotoxicity for ADC were determined for normalized data using logistic non-linear regression analysis with GraphPad Prism software version 10.2.1. Curve fits were constrained as needed to a bottom of greater than 0% viability and a top of less than 100% viability.
